# Supplementary material for: Network-based clustering unveils interconnected landscapes of genomic and clinical features across myeloid malignancies
Source: Nat Commun. 2025 Apr 30;16:4043. doi: 10.1038/s41467-025-59374-1 (PMC12041249; doi:10.1038/s41467-025-59374-1)
Supplement: Supplementary file 1 — Supplementary Information [file 41467_2025_59374_MOESM1_ESM.pdf]

Supplementary material: Network-based clustering unveils interconnected landscapes of genomic and clinical features across myeloid malignancies

Fritz Bayer[1,2], Marco Roncador[1,2], Giusi Moffa[3], Kiyomi Morita[4], Koichi Takahashi[4,5], Niko Beerenwinkel[1,2], and Jack Kuipers[1,2,*]

[1]Department of Biosystems Science and Engineering, ETH Zurich, Schanzenstrasse 44, 4056 Basel, Switzerland
[2]SIB Swiss Institute of Bioinformatics, Schanzenstrasse 44, 4056 Basel, Switzerland
[3]Department of Mathematics and Computer Science, University of Basel, Basel, Switzerland
[4]Department of Leukemia, The University of Texas MD Anderson Cancer Center, Houston, TX, USA
[5]Department of Genomic Medicine, The University of Texas MD Anderson Cancer Center, Houston, TX, USA
[*]Correspondence: jack.kuipers@bsse.ethz.ch

Supplementary Tables

Table S1. Summary table of patient characteristics.

Characteristic	All	AML	MDS	CMML	MPN
Number of patients	1323	543	492	118	170
Median age (years)	66	62	69	71	59
Range of age (years)	18-94	18-94	20-91	51-92	18-85
Number of males	792	296	324	87	85
Number of females	531	247	168	31	85

Table S2. Summary of variables included in our patient cohort. Only genes with a prevalence above 1% are retained, while the clinical variable that does not display differences across the disease types is excluded.

Variable type	Variable name
Demographic Variables	Age, Sex
Clinical Information	Cancer type, ANC, AMC, WBC, PLT, HGB, PB Blast, BM Blast, Mono, Gran, Auer Rod, Dysplasias
Mutations	*ASXL1, BCOR, BCORL1, CALR, CBL, CEBPA, CREBBP, CUX1, DDX41, DNMT3A, ETV6, EZH2, FLT3, GATA2, IDH1, IDH2, JAK2, KDM6A, KIT, KRAS, MPL, NF1, NPM1, NRAS, PHF6, PTPN11, RAD21, RUNX1, SETBP1, SF3B1, SRSF2, STAG2, SUZ12, TET2, TP53, U2AF1, WT1, ZRSR2*
Cytogenetics	-5/5q, -7/7q, +8/8q, -Y, +11/11q, inv16, t(8;21), -20/20q
Excluded variables	ALC

Table S3. Summary table of survival analysis by each cluster and cancer type. Groups with fewer than 10 individuals are omitted for clarity.

Cluster	Cancer type	Records	Events	RMST	SE(RMST)	Median OS	95LCL	95UCL
A	Overall	191	94	5.4	0.7	3.2	2.8	3.9
B	Overall	175	113	4.9	0.6	2.3	2.0	3.2
C	Overall	98	75	3.6	0.7	1.4	1.3	1.6
D	Overall	182	165	1.0	0.1	0.8	0.6	0.9
E	Overall	173	88	6.4	0.5	4.7	2.9	
F	Overall	158	21	14.4	1.1		14.2	
G	Overall	98	42	6.7	1.0	4.6	4.0	7.0
H	Overall	121	62	6.1	0.5	3.9	2.7	
I	Overall	127	65	5.1	0.7	3.6	2.8	5.4
A	AML	10	7	5.1	2.5	1.1	0.8	
A	MDS	174	85	5.4	0.7	3.2	2.8	4.0
B	AML	120	79	5.3	0.8	2.4	2.1	3.5
B	MDS	54	33	3.8	0.9	2.0	1.8	3.3
C	AML	47	38	3.8	1.0	1.3	1.0	1.5
C	CMML	19	13	3.0	0.6	1.8	1.6	
C	MDS	27	22	2.4	1.0	1.3	1.1	1.7
D	AML	72	68	1.0	0.1	0.6	0.4	1.0
D	MDS	109	96	1.4	0.3	0.8	0.7	1.0
E	AML	170	87	6.4	0.5	4.7	2.7	
F	MPN	151	19	14.7	1.1		14.2	
G	CMML	16	4	3.7	0.4	4.5	4.0	
G	MDS	73	34	6.4	1.1	4.6	3.7	7.0
G	MPN	8	3	10.7	3.5	7.5	7.5	
H	AML	118	61	6.1	0.5	3.9	2.7	
I	CMML	75	39	4.5	0.5	3.7	2.9	5.5
I	MDS	46	22	4.7	0.7	5.0	2.6	

Table S4. Likelihood ratio for different variations of the algorithm after accounting for clinical and histopathological covariates in the survival analysis of the pan-myeloid data (Likelihood ratio test from the adjusted Cox proportional hazards regression, without multiple testing corrections). First, the table shows the impact of the covariate-adjustment on the clustering compared to simply concatenating the data (without covariate-adjustment) or only using the mutational data. Second, the table shows the impact on the clustering of not using the STRING database as a prior for the network learning (last row).

Method	Corrected likelihood ratio	P-value
CANclust	**106.8**	9.1×10^{-42}
CANclust (without covariate-adjustment)	101.7	1.3×10^{-39}
CANclust (mutations only)	95.3	6.2×10^{-37}
CANclust (without STRING)	102.0	9.6×10^{-40}

Table S5. Likelihood ratio for an integrative clustering of AML and MDS combined (with 8 clusters since one cluster in the pan-myeloid analysis is predominantly MPN), compared to treating the two cancer types separately (4 clusters each). (Likelihood ratio test from the adjusted Cox proportional hazards regression, without multiple testing corrections).

Method	AML & MDS	Corrected likelihood ratio	P-value
CANclust	combined	**96.6**	3.2×10^{-38}
CANclust	separate	88.0	1.3×10^{-34}

Table S6. Likelihood ratio for our subgroups compared to those generated by the ELN2022 and IPSS-M risk scores. The table includes corrected likelihood ratios, which account for age, sex, and cancer type in the survival analysis (Likelihood ratio test from the adjusted Cox proportional hazards regression, without multiple testing corrections). The final two rows offer further corrections, incorporating the effects of the aforementioned risk scores into the survival analysis. In our cluster analysis and risk score calculations, we did not include certain features due to their absence in our dataset. Specifically, the following were excluded: complex karyotype, -17/del(17p), *TP53*$^{\text{multi-hit}}$, *GNB1, PPM1D, PRPF8*, t(9;11), t(6;9), t(9;22), inv(3), and i(17).

Grouping	Cancer type	Corrected likelihood ratio	Likelihood ratio	P-value
CANclust	AML	**29.2**	**165.1**	9.0×10^{-10}
ELN2022	AML	16.9	140.6	4.2×10^{-5}
CANclust	MDS	**76.4**	**154.4**	5.2×10^{-29}
IPSS-M	MDS	53.9	114.1	1.1×10^{-19}
CANclust (beyond ELN2022)	AML	21.2	182.9	1.2×10^{-6}
CANclust (beyond IPSS-M)	MDS	53.9	221.8	1.1×10^{-19}

Table S7. Summary table of survival analysis by each risk classification and cancer type.

Risk	Risk score	Records	Events	RMST	SE(RMST)	Median OS	95LCL	95UCL
Adverse	ELN2022 (AML)	256	198	3.3	0.4	1.4	1.1	1.5
Favorable	ELN2022 (AML)	215	102	6.9	0.4	6.8	3.9	
Intermediate	ELN2022 (AML)	72	45	5.8	0.9	1.9	1.3	3.5
Very High	IPSS-M (MDS)	56	39	1.9	0.2	1.5	1.1	1.9
High	IPSS-M (MDS)	112	77	2.8	0.6	1.4	1.3	1.8
Moderate High	IPSS-M (MDS)	81	62	2.0	0.2	1.5	1.1	2.0
Moderate Low	IPSS-M (MDS)	88	56	2.8	0.3	1.8	1.4	3.0
Low	IPSS-M (MDS)	130	57	6.2	0.6	4.6	3.8	6.5
Very Low	IPSS-M (MDS)	25	3	12.6	1.8		4.4	

Table S8. Likelihood ratio for our subgroups compared to those generated by the ELN2022 and IPSS-M risk scores on the validation cohort. The table includes corrected likelihood ratios, which account for age, sex, and cancer type in the survival analysis (Likelihood ratio test from the adjusted Cox proportional hazards regression, without multiple testing corrections). The final two rows offer further corrections, incorporating the aforementioned risk scores as predictors in the survival analysis. In our cluster analysis and risk score calculations, we did not include certain features due to their absence in our dataset. Specifically, the following were excluded: complex karyotype, -17/del(17p), *TP53*$^{\text{multi-hit}}$, *GNB1, PPM1D, PRPF8*, t(9;11), t(6;9), t(9;22), inv(3), and i(17).

Grouping	Cancer type	Corrected likelihood ratio	Likelihood ratio	P-value
CANclust	AML	**73.9**	**1057.2**	5.2×10^{-28}
ELN2022	AML	24.5	958.5	6.2×10^{-8}
CANclust	MPN	**19.7**	**81.1**	4.3×10^{-6}
IPSS-M	MPN	17.5	76.8	2.7×10^{-5}
CANclust (beyond ELN2022)	AML	57.5	1073.6	3.5×10^{-21}
CANclust (beyond IPSS-M)	MDS	13.4	103.5	7.7×10^{-4}

Supplementary Figures

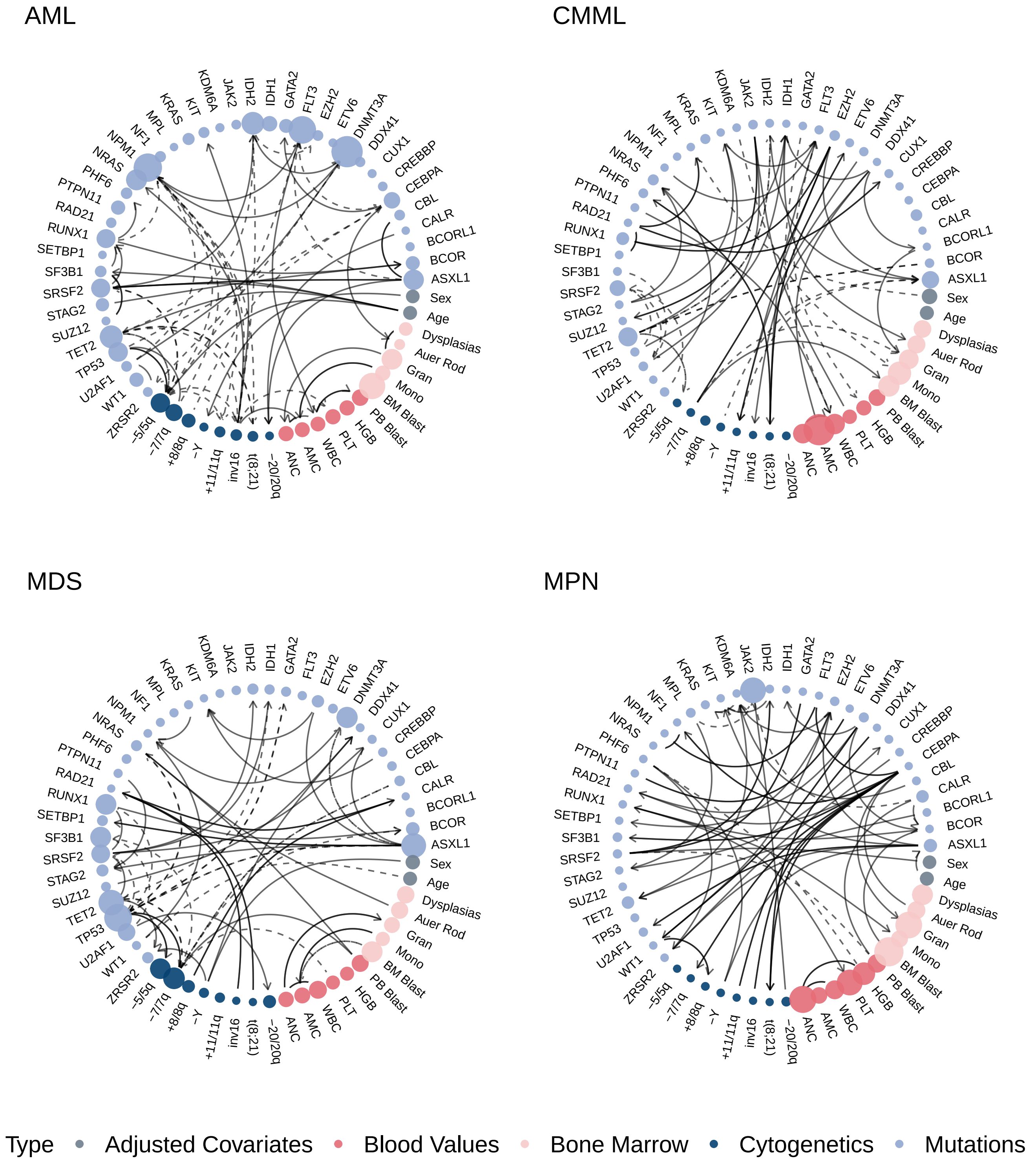

Figure S1. Cancer-specific networks, reproducing Figure 2 of the main text for the full set of variables. Genomic features are illustrated in blue, blood and bone marrow values in red and demographic variables in grey. The node size for mutations and cytogenetic variables corresponds to their frequency within each cluster, with more frequently mutated genes appearing as larger nodes. Conversely, the node size of continuous variables gets larger when the values of those variables are higher. Solid edges represent positive correlation while dashed edges represent negative correlation. AML: acute myeloid leukemia, CMML: chronic myelomonocytic leukemia, MDS: myelodysplastic syndromes, MPN: myeloproliferative neoplasms. AMC: absolute monocyte count, ANC: absolute neutrophil count, BM: bone marrow, HGB: hemoglobin, PB: peripheral blood, PLT: platelet, WBC: white blood count. Source data are provided as a Source Data file.

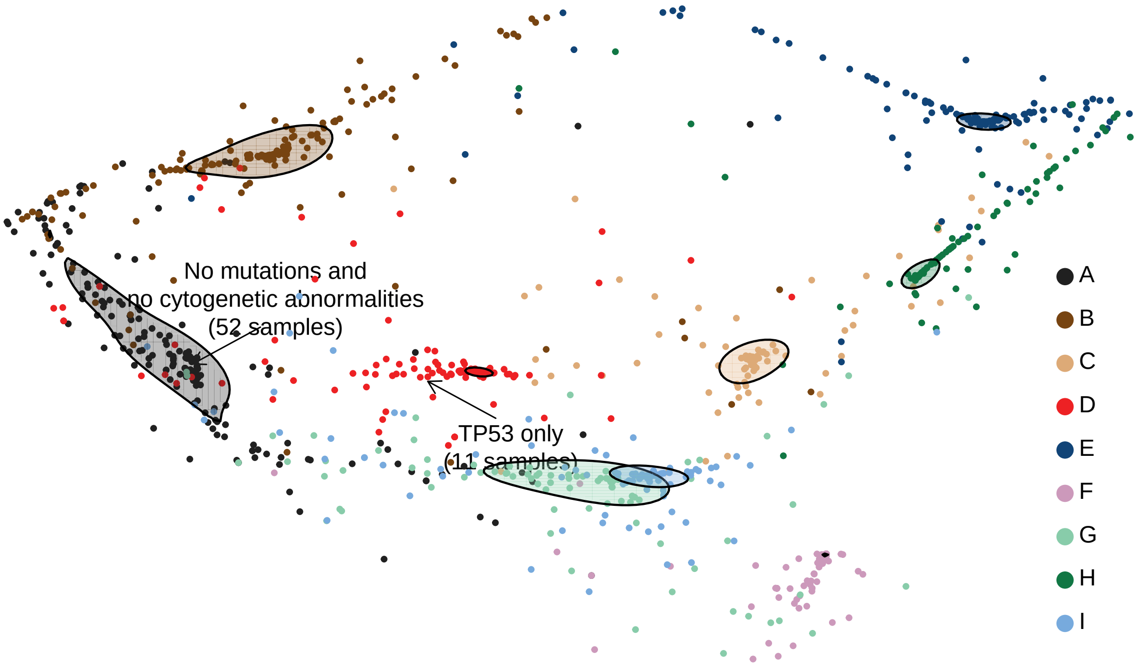

(a) Density plot of the different clusters coloured by cluster association.

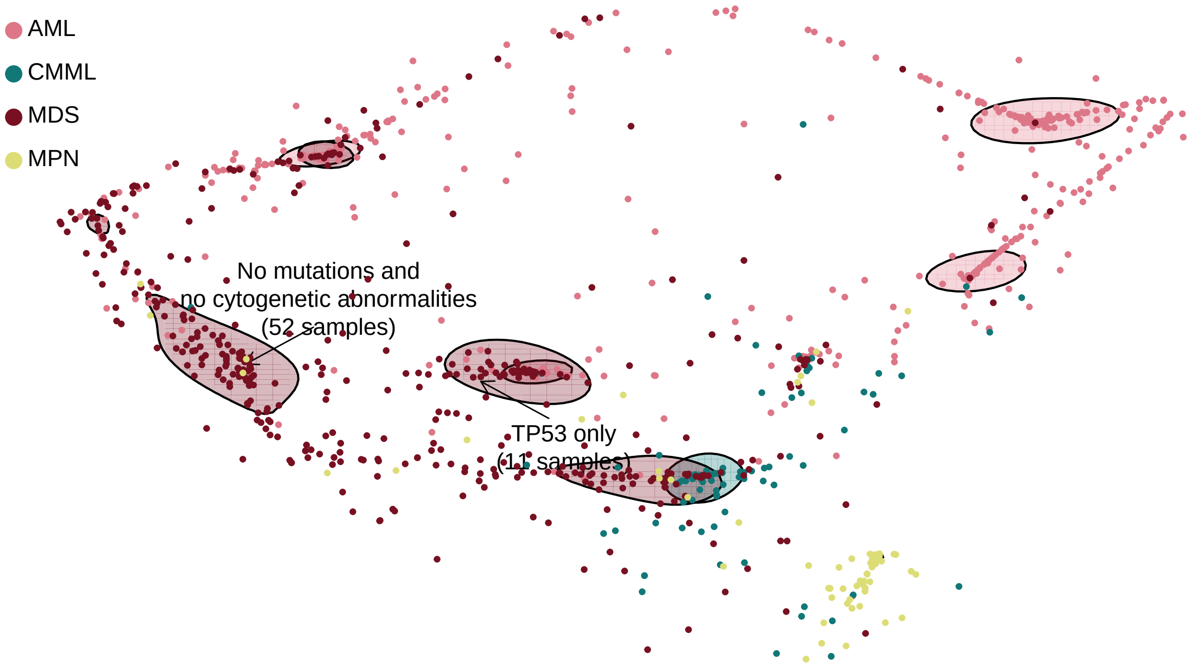

(b) Density plot of the different clusters coloured by cancer type.

Figure S2. Enlarged version of Figure 3a,b of the main text visualizing in 2D how well the patient samples fit to each cluster-specific Bayesian network. Each dot represents a patient and is coloured by its corresponding cluster. Solid shapes depict contours that collectively encompass 50% of the corresponding cluster (a) or cancer type (b). AML: acute myeloid leukemia, CMML: chronic myelomonocytic leukemia, MDS: myelodysplastic syndromes, MPN: myeloproliferative neoplasms. Source data are provided as a Source Data file.

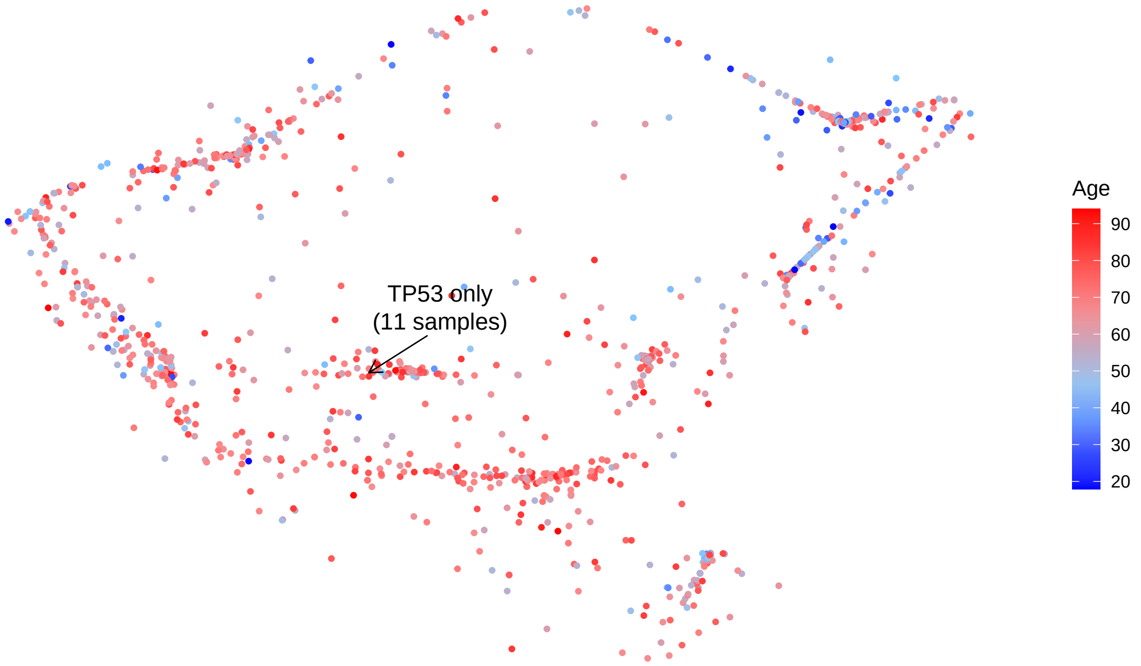

(a) Density plot of the different clusters coloured by cluster association.

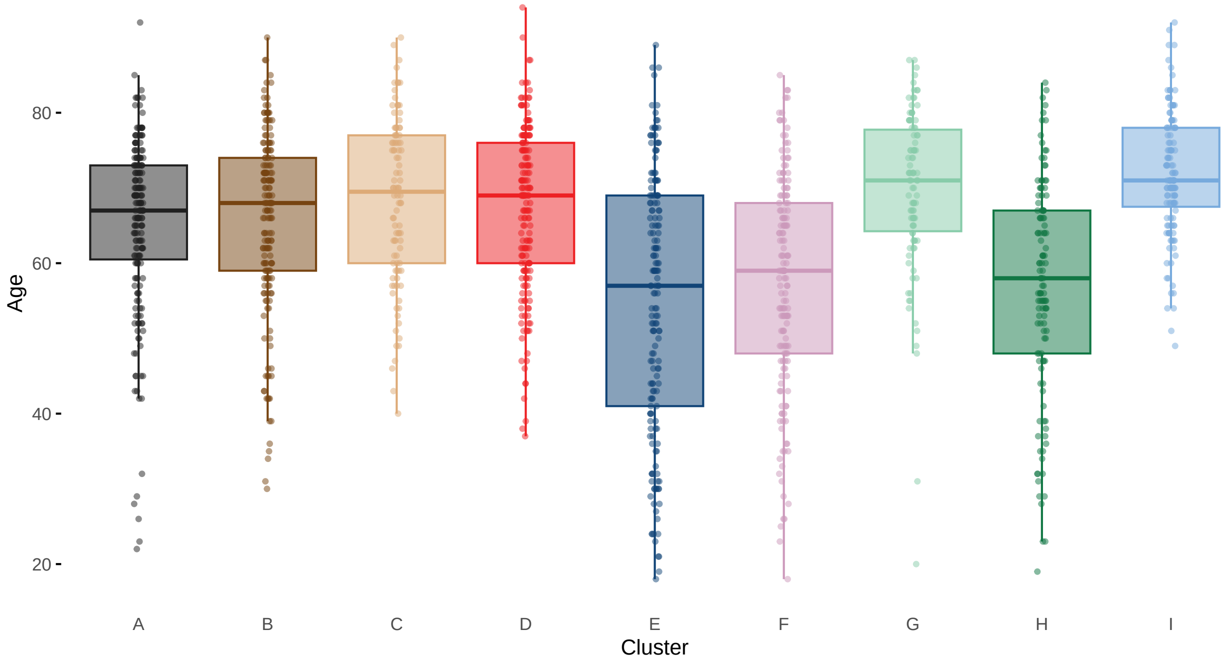

(b) Age distribution across clusters.

Figure S3. The visualization of Supplementary Figure S2 coloured by the age of each patient (a), and the distribution of ages per cluster (b).

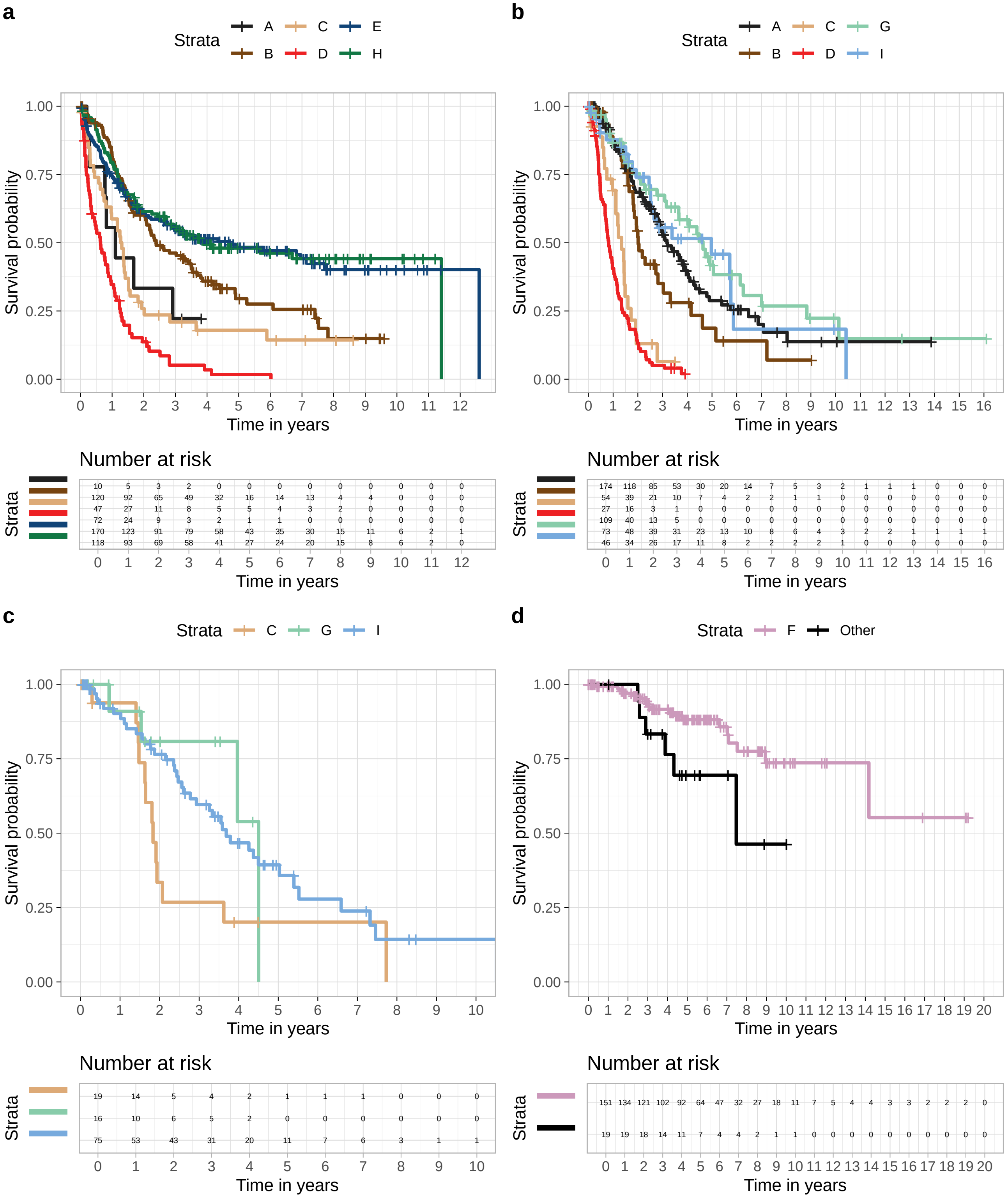

Figure S4. Kaplan-Meier survival curves for AML (a), MDS (b), CMML (c), and MPN (d) patients, stratified by cluster. Groups with fewer than 10 individuals are omitted for clarity. In MPN, due to only one cluster with more than 10 individuals, other clusters are grouped together. AML: acute myeloid leukemia, CMML: chronic myelomonocytic leukemia, MDS: myelodysplastic syndromes, MPN: myeloproliferative neoplasms.

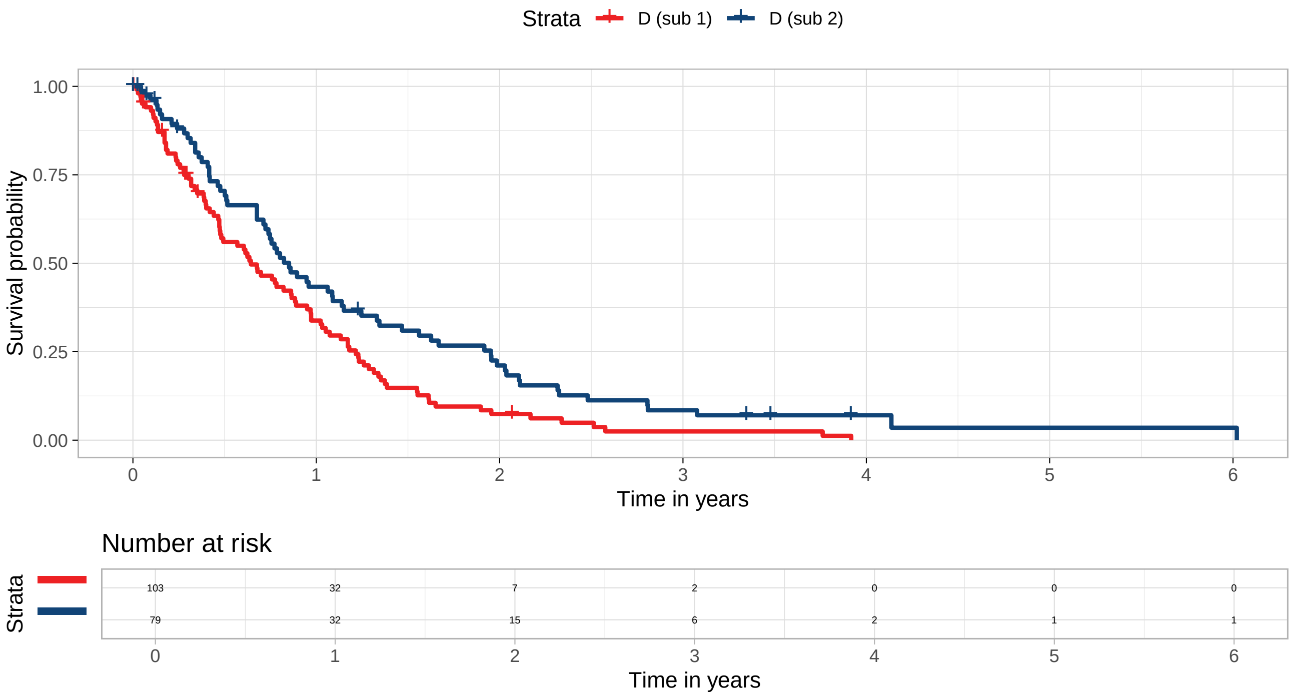

Figure S5. Kaplan-Meier survival curves for cluster D, split into two subgroups based on their probability of belonging to cluster A. A cut-off of $p_A = 10^{-3}$ was used to split the two groups. D (sub 2) shows patients from cluster D, with higher similarity to cluster A, while D (sub 1) shows patients with a lower similarity to cluster A. The corrected LR is 3.4 (13.4 uncorrected LR) and the p-value is 0.0094. (Likelihood ratio test from the adjusted Cox proportional hazards regression, without multiple testing corrections).

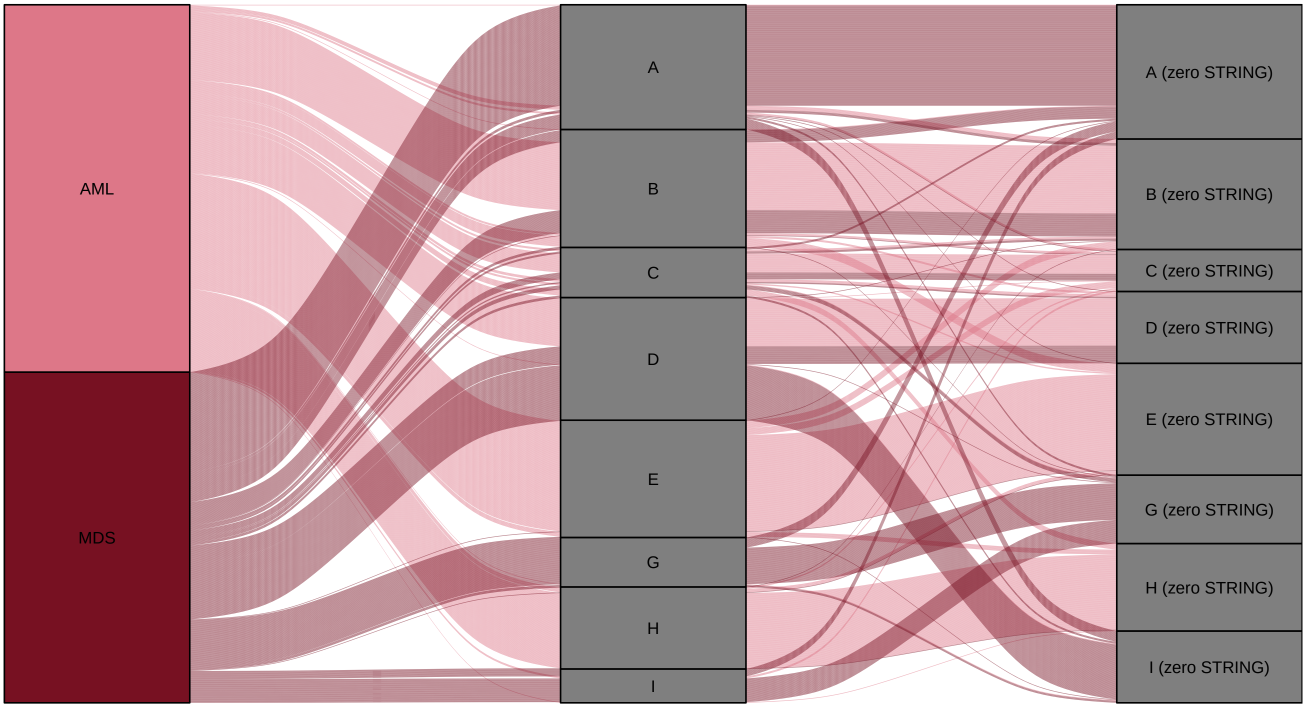

Figure S6. Sankey plot comparing the cluster assignments that were learned with and without including information from the STRING database as a prior for the network learning. The final clustering, informed by the STRING database, is labelled *A - I*. For comparison, cluster assignments that were obtained without incorporating information from the STRING database in the learning process are labelled *A (zero STRING) - I (zero STRING)*. Cluster *F* is not displayed as it primarily contains MPN samples. AML: acute myeloid leukemia, MDS: myelodysplastic syndromes, MPN: myeloproliferative neoplasms. Source data are provided as a Source Data file.

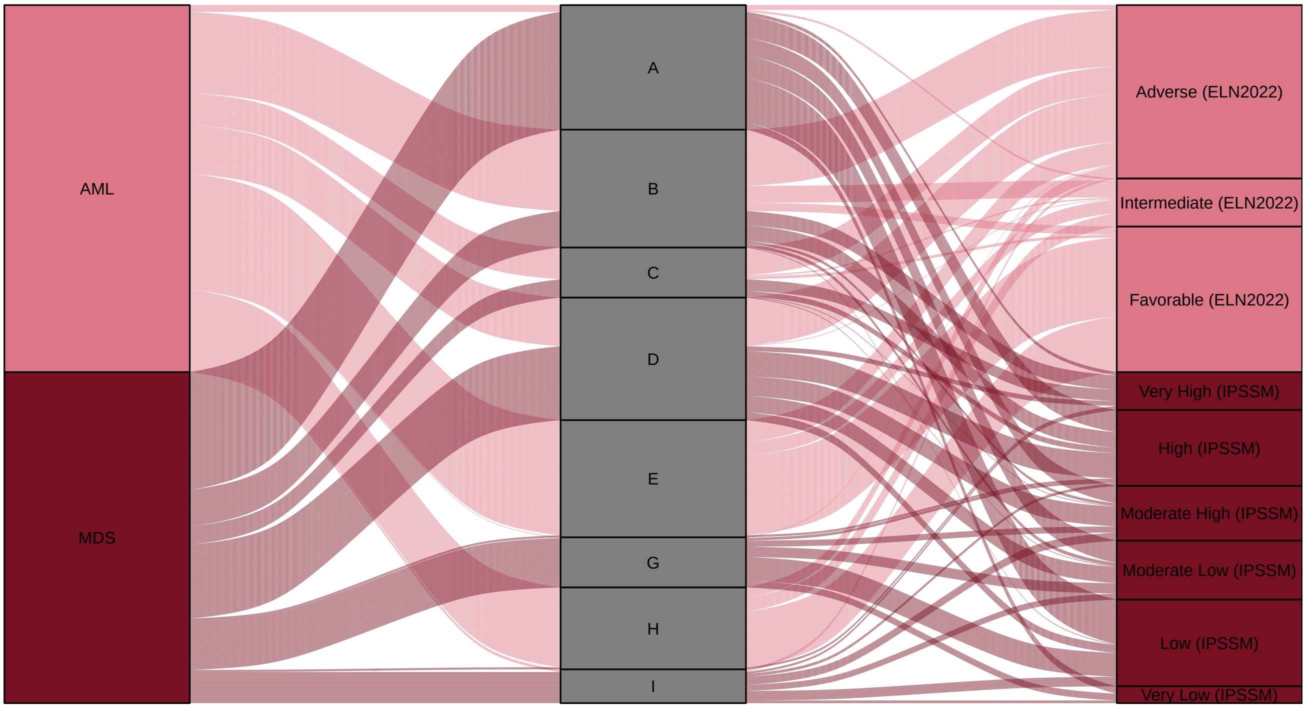

Figure S7. Sankey plot of AML and MDS risk scores. Cluster F is not displayed as it primarily contains MPN samples. AML: acute myeloid leukemia, MDS: myelodysplastic syndromes, MPN: myeloproliferative neoplasms. Source data are provided as a Source Data file.

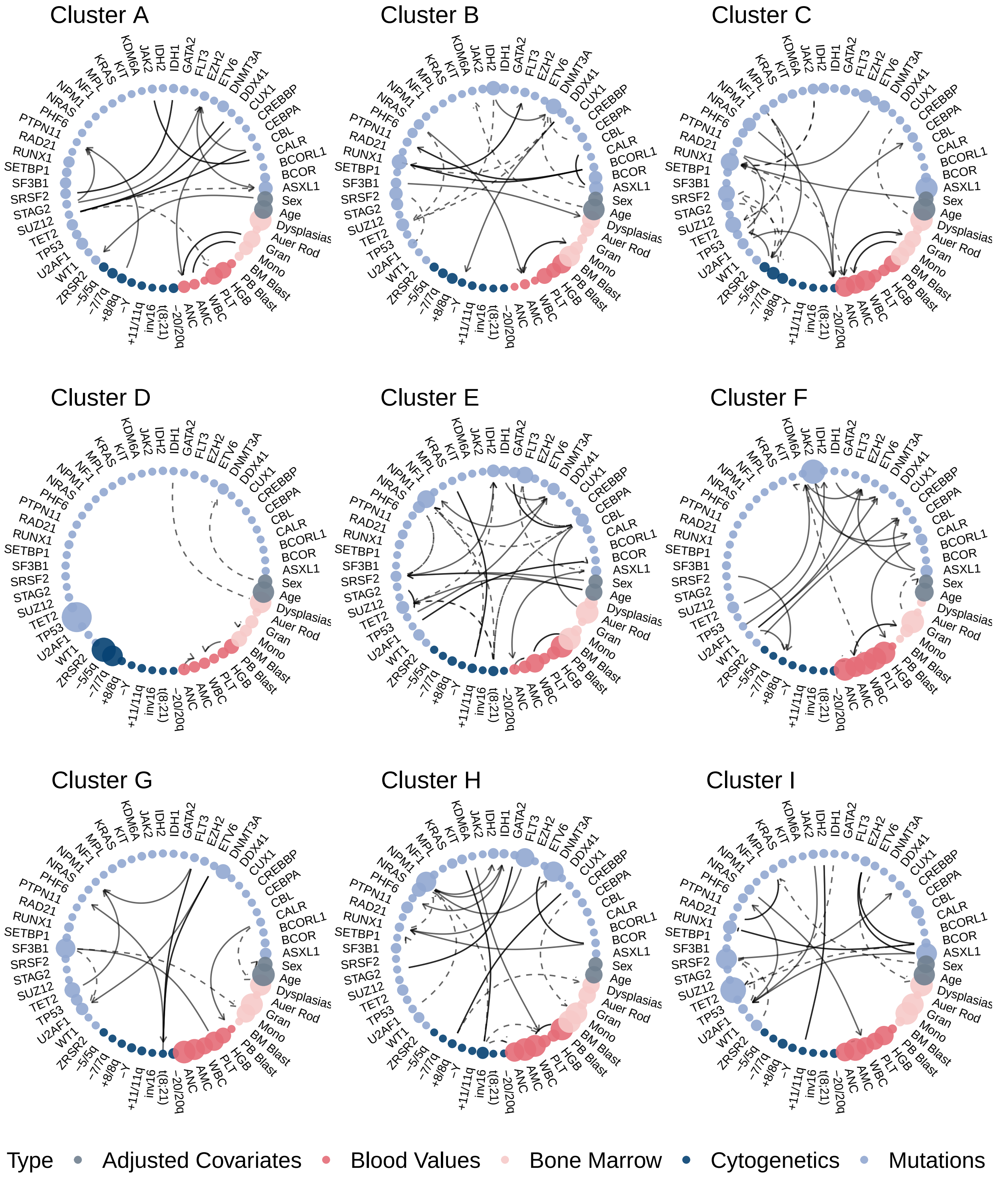

Figure S8. Cluster-specific networks of the clusters A-I, reproducing Figure 5 of the main text for the full set of variables. Genomic features are illustrated in blue, blood and bone marrow values in red and demographic variables in grey. The node size for mutations and cytogenetic variables corresponds to their frequency within each cluster, with more frequently mutated genes appearing as larger nodes. Conversely, the node size of continuous variables gets larger when the values of those variables are higher. Solid edges represent positive correlation while dashed edges represent negative correlation. AMC: absolute monocyte count, ANC: absolute neutrophil count, BM: bone marrow, HGB: hemoglobin, PB: peripheral blood, PLT: platelet, WBC: white blood count. Source data are provided as a Source Data file.

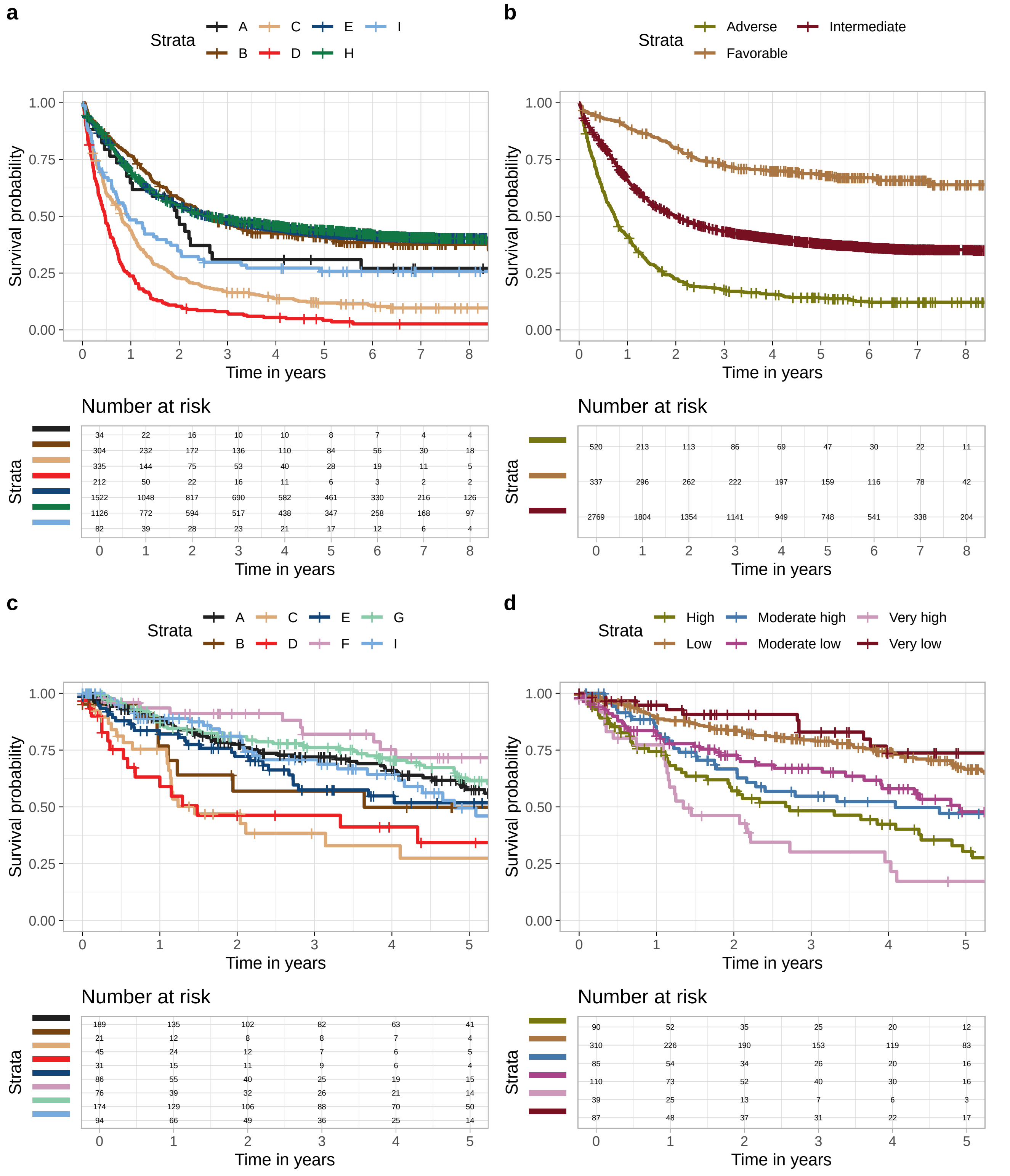

Figure S9. Kaplan-Meier survival curves for the validation cohorts of 3626 AML patients[1] and 875 MDS patients[2] comparing cluster assignments and ELN2022 and IPSS-M risk classifications in AML and MDS, respectively. Displayed are the survival probabilities for distinct clusters in AML patients (a) and their survival outcomes stratified by ELN2022 risk classification (b). Corresponding analyses for MDS include survival probabilities across distinct clusters (c) and according to IPSS-M risk classification (d). Groups with fewer than 10 individuals are omitted for clarity. AML: acute myeloid leukemia, MDS: myelodysplastic syndromes.

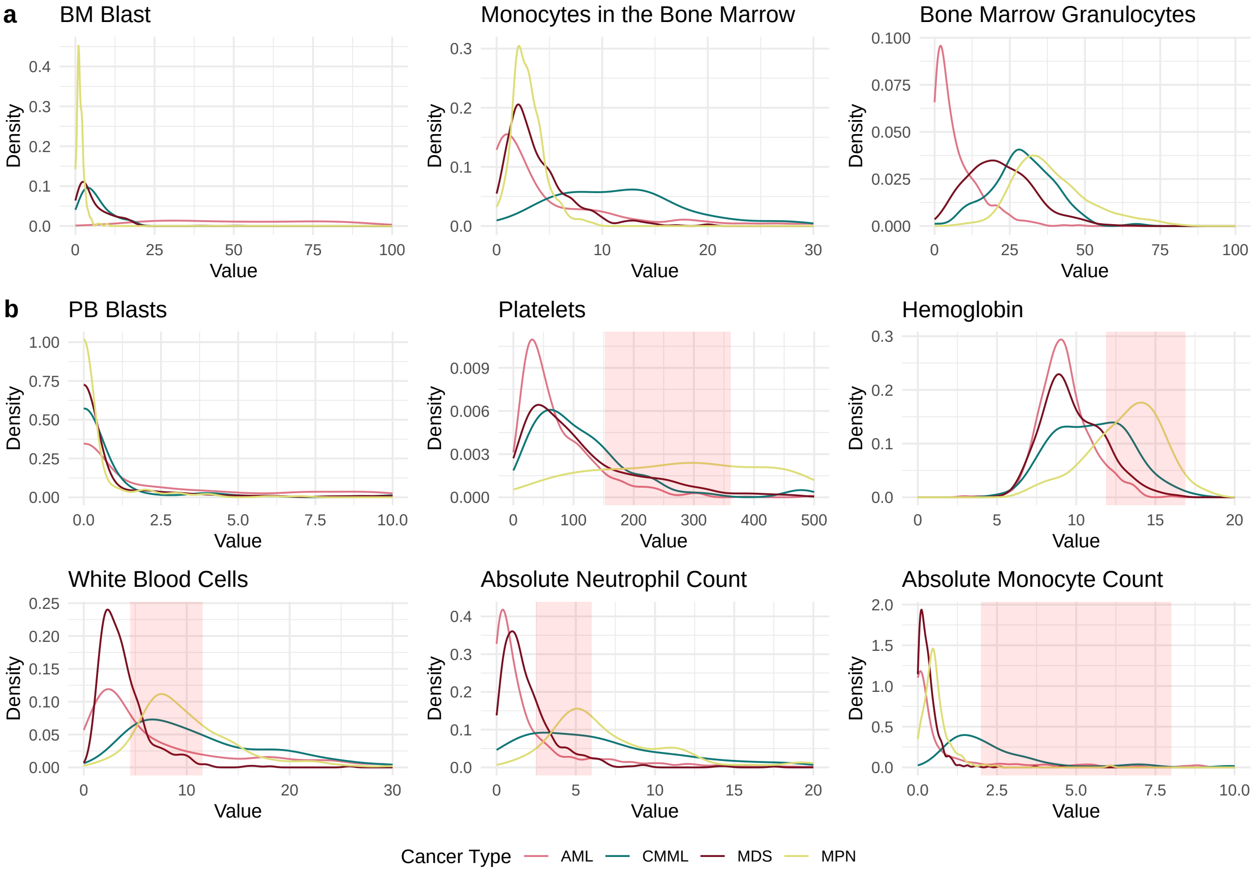

Figure S10. Distribution of the continuous variables in bone marrow (a) and blood (b). Displayed are the kernel density plots of the distributions for each cancer type. The normal range observed in healthy individuals is displayed in transparent red. BM: bone marrow, PB: peripheral blood.

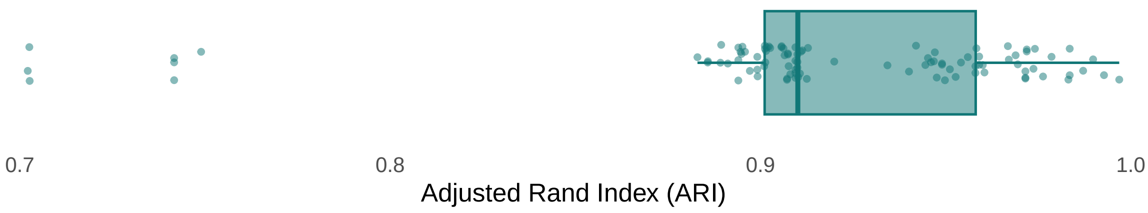

Figure S11. Boxplot showing the adjusted Rand index across different replications of the clustering procedure for different random seeds. Source data are provided as a Source Data file.

A Supplementary Methods

A.1 Adjusting for Covariates

We want to cluster the mutational profiles to a number of K individual clusters. We assume that the mutational profiles X_V can be represented by the probability distributions of K Bayesian networks, and that the cluster-dependent clinical covariates X_C affect mutations via directed edges from covariates to mutations.

In the Bayesian network mixture model, we can consider the following three clustering variations: covariate-adjusted clustering, joint clustering of mutational profiles and clinical covariates, and clustering of mutational profiles only.

For the covariate-adjusted clustering, the membership probability function is defined as

$$\tilde{\phi}(X_V \mid k) = \frac{\gamma_k \cdot P\left(X_V \mid X_C, \hat{\mathscr{G}}_k, \hat{\theta}_k\right)}{\sum_{k'=1}^{K} \gamma_{k'} \cdot P\left(X_V \mid X_C, \hat{\mathscr{G}}_{k'}, \hat{\theta}_{k'}\right)} . \tag{1}$$

For the clustering of mutational profiles and clinical covariates, it is

$$\phi(X_V, X_C \mid k) = \frac{\gamma_k \cdot P\left(X_V, X_C \mid \hat{\mathscr{G}}_k, \hat{\theta}_k\right)}{\sum_{k'=1}^{K} \gamma_{k'} \cdot P\left(X_V, X_C \mid \hat{\mathscr{G}}_{k'}, \hat{\theta}_{k'}\right)} . \tag{2}$$

For the clustering of mutational profiles, it is

$$\phi(X_V \mid k) = \frac{\gamma_k \cdot P\left(X_V \mid \hat{\mathscr{G}}_k, \hat{\theta}_k\right)}{\sum_{k'=1}^{K} \gamma_{k'} \cdot P\left(X_V \mid \hat{\mathscr{G}}_{k'}, \hat{\theta}_{k'}\right)} . \tag{3}$$

If we assume that the probability distribution of the cluster-independent covariates X_C is constant across different clusters, i.e. $\forall k, k' \in \{1, ..., K\} : P(X_C \mid k) = P(X_C \mid k')$ and if the Bayesian network parameters $(\hat{\mathscr{G}}_k, \hat{\theta}_k)$ are known, then the membership probability functions defined in Equation 1 and Equation 2 are identical:

$$\begin{aligned}
\phi(X_V, X_C \mid k) &= \frac{\gamma_k \cdot P\left(X_V, X_C \mid \hat{\mathscr{G}}_k, \hat{\theta}_k\right)}{\sum_{k'=1}^{K} \gamma_{k'} \cdot P\left(X_V, X_C \mid \hat{\mathscr{G}}_{k'}, \hat{\theta}_{k'}\right)} \\
&= \frac{\gamma_k \cdot P\left(X_C \mid \hat{\mathscr{G}}_k, \hat{\theta}_k\right) \cdot P\left(X_V \mid X_C, \hat{\mathscr{G}}_k, \hat{\theta}_k\right)}{\sum_{k'=1}^{K} \gamma_{k'} \cdot P\left(X_C \mid \hat{\mathscr{G}}_{k'}, \hat{\theta}_{k'}\right) \cdot P\left(X_V \mid X_C, \hat{\mathscr{G}}_{k'}, \hat{\theta}_{k'}\right)} \\
&= \frac{\gamma_k \cdot P\left(X_V \mid X_C, \hat{\mathscr{G}}_k, \hat{\theta}_k\right)}{\sum_{k'=1}^{K} \gamma_{k'} \cdot P\left(X_V \mid X_C, \hat{\mathscr{G}}_{k'}, \hat{\theta}_{k'}\right)} = \tilde{\phi}(X_V \mid k)
\end{aligned}$$

Following Blackwell-Rao theorem, this implies that the variance of Equation 1 is lower than the variance in Equation 2, since the estimation of $P(X_C \mid \hat{\mathscr{G}}_k, \hat{\theta}_k)$ introduces further variance in the estimation. In addition, applying our causal knowledge about the direction of the causal pathways allows us to learn the DAGs $\hat{\mathscr{G}}_k$ more accurately, reducing the variance in $P(X_V \mid \hat{\mathscr{G}}_k, \hat{\theta}_k)$. If the covariates are excluded from the analysis (Equation 3), they act as hidden variables in the probability distribution of the mutations, leading to a lower accuracy in the cluster assignment.

We will now discuss two alternative scenarios to the assumption of a constant probability distribution of the covariates across the clusters. First, instead of being constant, the probability distribution of the covariates could depend on the clusters, i.e. $\exists k, k' \in \{1, ..., K\} : P(X_C \mid k) \neq P(X_C \mid k')$. In this case, the covariates would be cluster-dependent covariates that are informative for the cluster assignment. Thus, the covariates should be treated like cluster-dependent covariates without adjustments. Another scenario could be that the probability distribution of the covariates depends on a different grouping than the clusters $g \in \{1, ..., G\}$, i.e. $P(X_C \mid k, g) \neq P(X_C \mid k, g')$. In this case, adjusting for the covariates helps to remove the bias of the other grouping in the clustering of the mutations.

A.2 Algorithm

As outlined in Algorithm 1, we cluster the mutational profiles and clinical covariates using a mixture model. We use a Bernoulli mixture model to initialize the membership probabilities and learn the DAGs $\mathscr{G}_k$ with corresponding local probability

Algorithm 1: Covariate-Adjusted Clustering

Input: A matrix of variables X_V and a matrix of covariates X_C, pseudo-count hyperparameter χ and a cutoff limit ε
Output: Cluster membership probabilities $\tilde{\phi}(X_V)$ and respective Bayesian networks $(\mathscr{G}_k, \theta_k)$

Initialize membership probabilities $\tilde{\phi}(X_V \mid k)$
repeat
  $i \leftarrow 0$
  Create copy of membership probabilities $\tilde{\phi}_{\text{previous}}(X_V \mid k) \leftarrow \tilde{\phi}(X_V \mid k)$
  Learn the DAGs $\mathscr{G}_k$ given the membership probabilities $\tilde{\phi}(X_V \mid k)$
  repeat
    $i \leftarrow i+1$
    M-Step: Learn the parameters θ_k given $\mathscr{G}_k$ and $\tilde{\phi}(X_V \mid k)$
    E-Step: Update membership probability: $\tilde{\phi}(X_V \mid k) \leftarrow \frac{\chi+\gamma_k \cdot P\left(X_V|X_C,\hat{\mathscr{G}}_k,\hat{\theta}_k\right)}{\chi K+\sum_{k'=1}^{K}\gamma_{k'} \cdot P\left(X_V|X_C,\hat{\mathscr{G}}_{k'},\hat{\theta}_{k'}\right)}$
    Update the cluster weights γ_k: $\gamma_k \leftarrow \frac{\sum_{1=1}^{N}\tilde{\phi}(X_V|k)}{N}$
  until $i = 10$;
  Quantify change in membership probabilities $\delta \leftarrow \sum_{X_V}\left(\tilde{\phi}(X_V \mid k) - \tilde{\phi}_{\text{previous}}(X_V \mid k)\right)^2$
until $\delta < \varepsilon$;

distributions θ_k using the **BiDAG** package (GPL-3)[3]. Due to the high computational cost of the DAG structure search, we only relearn the DAGs $\mathscr{G}_k$ for every tenth update of the membership probabilities.

Our computations were performed on one CPU core of the AMD EPYC 7H12 processor (2.6 GHz nominal, 3.3 GHz peak) and 256 GB of DDR4 memory clocked at 3200 MHz. Code implementing our method is publicly available as a software package at `https://CRAN.R-project.org/package=clustNet`.

While our algorithm presents several advantages, it comes with the trade-off of higher computational cost compared to traditional clustering methods like k-means or Bernoulli mixture models. The computational cost scales notably with an increase in the number of variables. In our simulations, the computation time averaged 4.18 minutes over 20 repetitions with the following parameters: 4 clusters, 20 variables, 5 covariates, and 4800 samples. Clustering the mutational and clinical profiles from the pan-myeloid data (9 clusters, 59 variables, 13 covariates, 1323 samples), our method took 3.4 hours to converge. The clustering of the TCGA data (22 clusters, 201 variables, 24 covariates, 8085 samples) took 30.32 hours to converge. By parallelizing the structure learning step of the individual clusters, we were able to reduce the runtime of our algorithm by a factor of 1.6 for two clusters.

A.3 Evolutionary Perspective

Cancer progression can be described as the somatic evolution of mutations in the genome[4]. The occurrence of mutations is commonly modelled using mutation trees, in which the presence of one mutation makes another mutation more likely to occur[5]. This process of genetic progression creates mutational patterns of probabilistic relationships among the genes. Bayesian networks are a natural framework to model the mutational patterns that are created through somatic evolution[6]. Bayesian networks are a powerful tool to model the probabilistic relationships amongst random variables and allow for a natural integration of covariates. While an evolutionary tree describes possible orders in which the mutations can occur[5], a Bayesian network can describe more general probabilistic relationships amongst the mutations[7]. Note that the nodes in the mutation tree contain a list of mutated genes, whereas the nodes in the Bayesian network contain the genes themselves, which can either be mutated or not.

Besides Bayesian networks, there are various other probabilistic graphical models. The most popular models include tree graphical models and Markov random fields (undirected graphical models)[8]. However, mutational trees induce nodes with more than one parent, which can not be modelled by tree graphical models. On the other hand, undirected models can model evolutionary trees, but can not model the order in which the genes occur.

Clinical covariates such as sex and age are closely related to the mutations in specific genes[9,10]. Such dependencies can be efficiently modelled in the framework of Bayesian networks by edges from the covariates to the respective genes.

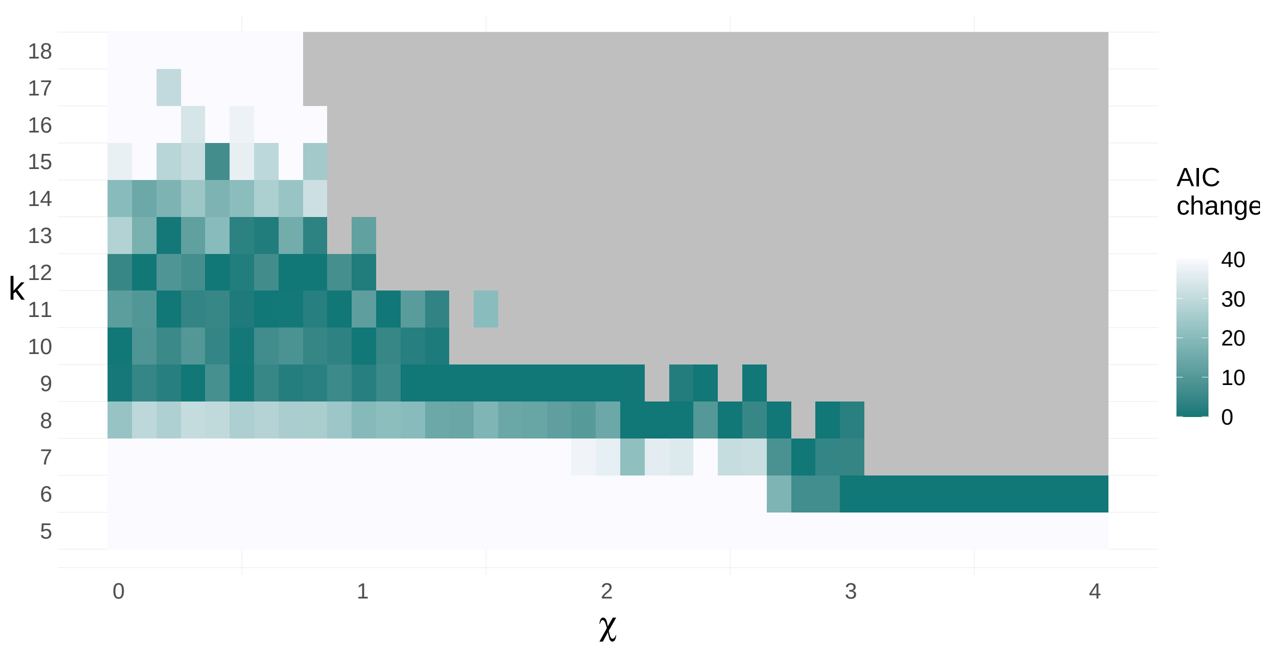

Figure S12. AIC (Akaike information criterion) change over different numbers of clusters. Grey areas mark the configurations where empty clusters were present. Source data are provided as a Source Data file.

A.4 Number of clusters

To determine the optimal number of clusters, we clustered the data using a range of cluster numbers and hyperparameter χ. We used the AIC (Akaike information criterion) for selecting the best-fitting number of clusters, with lower scores indicating better fits. In this analysis, we neglected the edges between nodes, which is equivalent to using a Bernoulli mixture model. We focused on mutations and cytogenetic variables to determine the number of clusters as we were interested in cluster associations based on genetic patterns. Including the clinical covariates would lead to a higher number of clusters, as these variables are not as sparse as the mutational and cytogenetic profiles. While a higher number of clusters may also yield meaningful cluster associations, it reduces the number of samples per cluster, resulting in lower certainty in the learned networks. Applying our method to a larger dataset may therefore allow learning a higher number of clusters.

Supplementary Figure S12 shows the AIC scores for different numbers of clusters and hyperparameters χ, where the difference to the lowest value is displayed; cluster assignments that contained empty clusters are marked in grey. We chose the cluster number nine, as it most frequently resulted in the lowest AIC score, which varied across the range of χ. For larger values of $\chi > 2$, the individual data points had a lower weight, leading to a smaller number of clusters.

B Simulations

For our setting, we consider a set of variables representing the mutation profiles of patient tumours, which we refer to as mutations, and a set of clinical covariates for each patient, which we refer to simply as covariates. To evaluate the performance of our covariate-adjusted clustering method, we benchmarked it against standard clustering methods over a range of different simulations, which were selected to cover typical pan-cancer applications. To account for our biological assumptions, we simulated data with cluster-specific probabilistic relationships and causal effects from covariates on the clustered variables. We simulated a Bayesian network for each cluster, including covariates as nodes with outgoing edges and constant conditional probability tables.

We compared our method against the following clustering algorithms: a Bayesian network mixture model (BNMM), k-means and a Bernoulli Mixture Model (BMM). Since these clustering algorithms do not adjust for covariates, we applied them once by adding the covariates to the clustered variables and once by excluding the covariates from the analysis. Code implementing our method and reproducible simulation benchmarks are available at `https://github.com/cbg-ethz/myeloid-clustering`.

Supplementary Figure S13 shows the benchmark results over different numbers of covariates, where clustering accuracy was assessed via the adjusted rand index (ARI). In the presence of covariates, the most accurate clustering performance is reached by our covariate-adjusted method. This is in line with our theoretical expectations, which removed the effect of the covariates

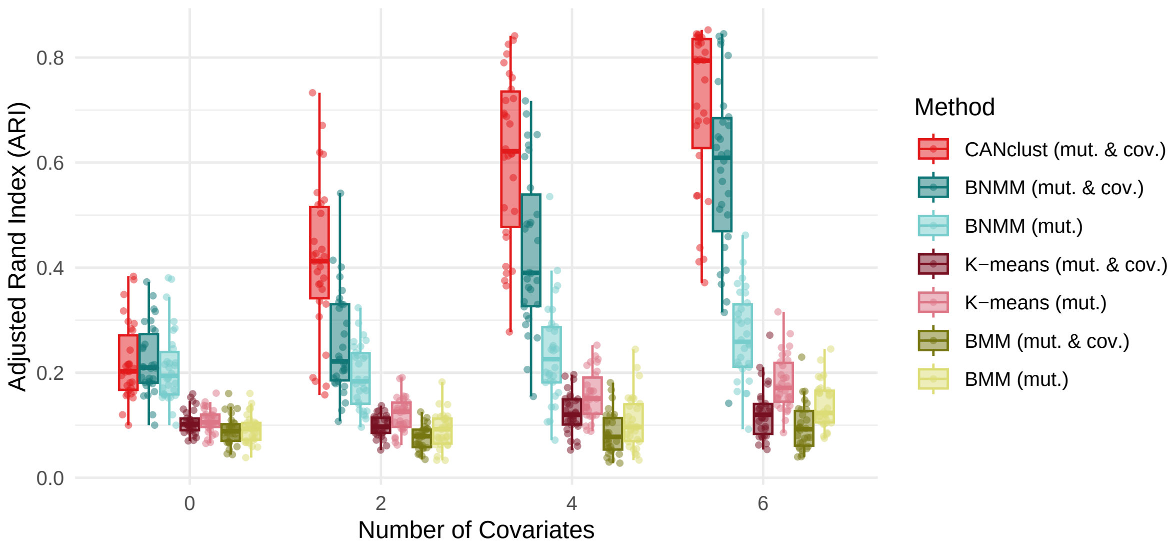

Figure S13. Simulation benchmark for an increasing number of covariates, comparing our covariate-adjusted network clustering algorithm (CANclust) against a Bayesian network mixture model (BNMM), k-means, and a Bernoulli mixture model (BMM). Standard clustering algorithms that do not adjust for covariates were applied twice: first, by including the covariates, and second, by excluding the covariates. Source data are provided as a Source Data file.

on the cluster membership probability while adjusting for their other effects. In general, the network-based clustering methods have a higher clustering accuracy, which reflects that these structures allow one to model probabilistic relationships among the variables. Supplementary Figure S13 shows that k-means and BMM perform better when the covariates are excluded from the analysis. This can be explained by the fact that these models assume independent probability distributions and hence including the covariates only increases the variance in the cluster assignment. In contrast, the performance of the BNMM improves when including the covariates, since their induced probabilistic relationships allow one to model the data more accurately.

To assess the performance of our covariate-adjusted algorithm in more detail over a range of different scenarios in our benchmark, we varied the following cluster-specific parameters: number of clusters K, number of variables N_V, number of covariates N_C, and number of samples per cluster n_k. While varying one parameter in our benchmark, all other parameters remained constant. The results of the different benchmarks are displayed in Supplementary Figure S14.

C Application to TCGA Pan-Cancer Data

We applied our clustering algorithm to a large-scale genomic dataset from the Cancer Genome Atlas (TCGA)[11], which includes the mutational profiles and clinical information of 8085 patients from 22 different cancer types. For each primary cancer type, we considered the 16 most significantly mutated genes, adding up to a total of 201 genes across all cancer types[7]. In addition, we included the clinical covariates age, sex, and cancer type in the analysis. Since the variables age, sex and tissue of origin have a downstream causal effect on the mutations, we adjusted for them in our covariate-adjusted clustering framework. This is different from our modelling of the pan-myeloid data where the cancer type can be a consequence of the genomic data and is hence excluded from the adjustment. The number of clusters was determined from the AIC for a range of different sizes.

While there is no ground truth cluster assignment in pan-cancer data, similarity in survival outcomes within the clusters is a proxy indicator of shared biological properties. We applied the Cox proportional hazards regression model and corrected for the effects of age, stage, and cancer type on the results since these are strong predictors of survival.

Using our method to cluster the mutational profiles based on their probabilistic relationships, we identified 22 cancer subgroups for which the survival probabilities are shown in Supplementary Figure S15. Each subgroup is associated with a distinct network, representing its subgroup-specific probabilistic relationships. Supplementary Table S9 shows the corrected likelihood ratio (LR) of the Cox proportional hazards regression model for different clustering algorithms, where we only considered network-based approaches due to their superior performance in the benchmark study. High corrected LRs indicate cluster assignments that are highly predictive of survival. The lowest corrected LR is reached by the BNMM that neglects the clinical covariates, highlighting the importance of integrating these when clustering mutational profiles. In contrast, the cancer subgroups found with our covariate-adjusted clustering method were most predictive in survival beyond clinical information ($\text{LR} = 46.6$, p-value $= 1.0 \times 10^{-10}$), confirming our theoretical expectations and simulation results.

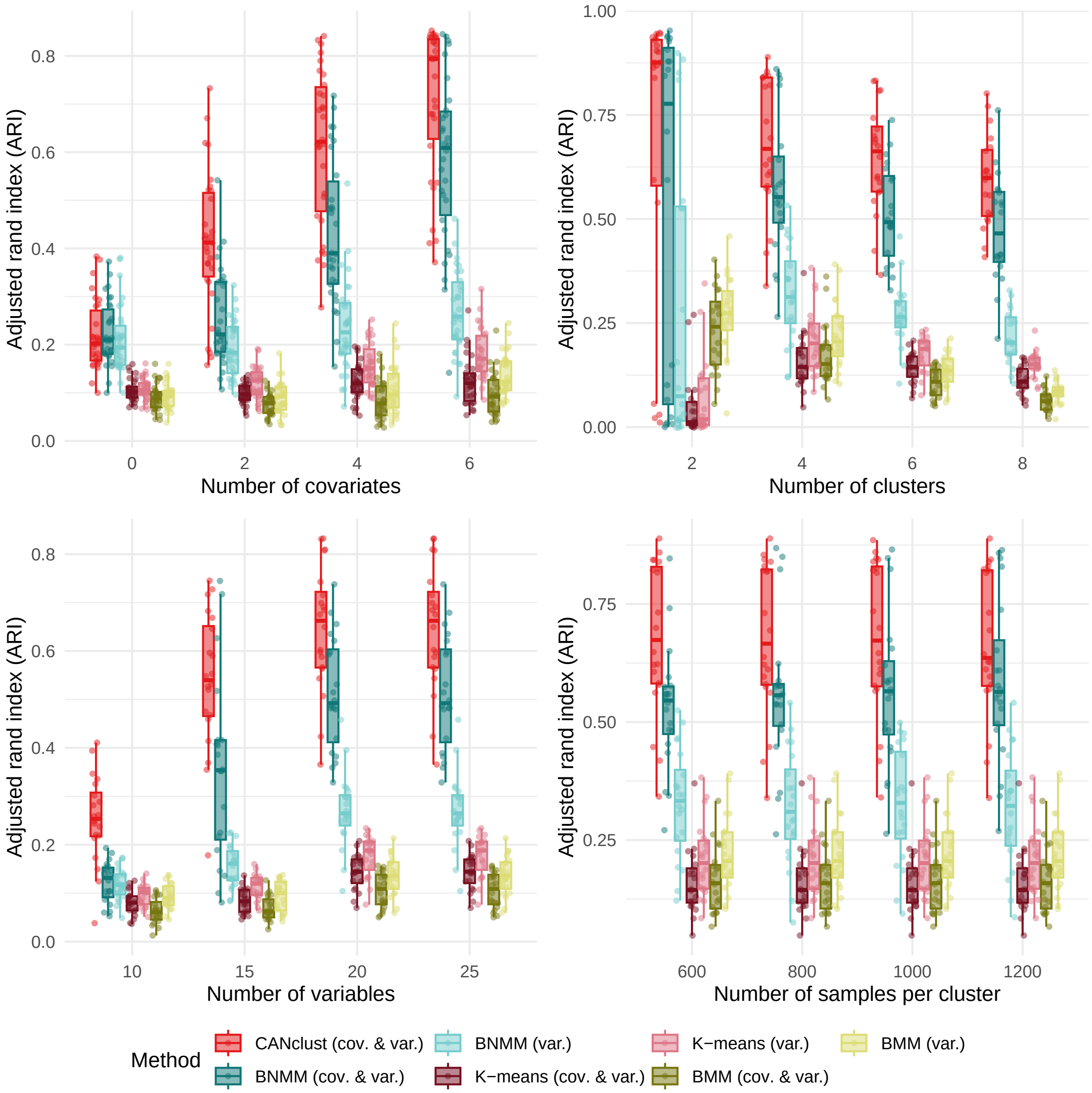

Figure S14. Simulation benchmark over a range of varied cluster-specific parameters including our covariate-adjusted network clustering algorithm (CANclust), a Bayesian network mixture model (BNMM), k-means, and a Bernoulli mixture model (BMM). Source data are provided as a Source Data file.

Table S9. Likelihood ratio for different clustering algorithms after accounting for clinical and histopathological covariates in the survival analysis of the TCGA data. (Likelihood ratio test from the adjusted Cox proportional hazards regression, without multiple testing corrections).

Method	Corrected likelihood ratio	P-value
CANclust	**46.6**	1.0×10^{-10}
BNMM (mut. & cov.)	43.8	4.0×10^{-10}
BNMM (mut.)	34.4	5.6×10^{-07}

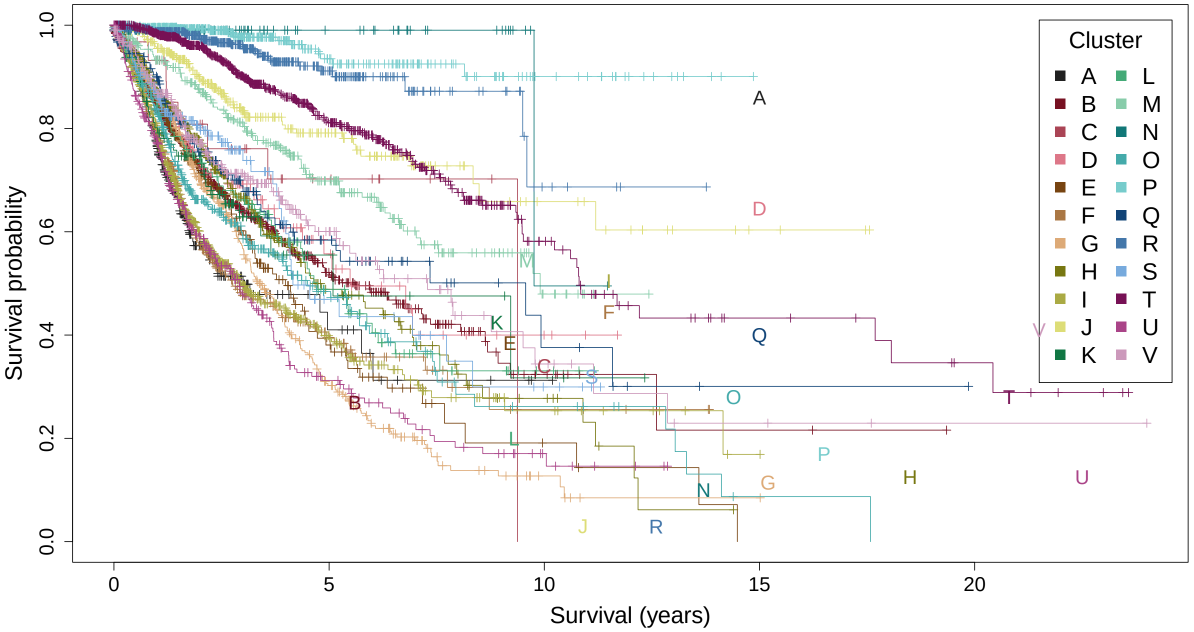

Figure S15. Survival probabilities of our cancer subgroups learned using network-based clustering, labelled A-V. Source data are provided as a Source Data file.

References

1. Tazi, Y. *et al.* Unified classification and risk-stratification in acute myeloid leukemia. *Nat. Commun.* **13**, 4622 (2022).

2. Kewan, T. *et al.* Molecular patterns identify distinct subclasses of myeloid neoplasia. *Nat. Commun.* **14**, 3136 (2023).

3. Suter, P., Kuipers, J., Moffa, G. & Beerenwinkel, N. Bayesian structure learning and sampling of Bayesian networks with the R package BiDAG. *J. Stat. Softw.* **105**, 1–31 (2023).

4. Greaves, M. & Maley, C. C. Clonal evolution in cancer. *Nature* **481**, 306–313 (2012).

5. Beerenwinkel, N., Schwarz, R. F., Gerstung, M. & Markowetz, F. Cancer evolution: mathematical models and computational inference. *Syst. Biol.* **64**, e1–e25 (2015).

6. Friedman, N., Linial, M., Nachman, I. & Pe'er, D. Using Bayesian networks to analyze expression data. *J. Comput. Biol.* **7**, 601–620 (2000).

7. Kuipers, J. *et al.* Mutational interactions define novel cancer subgroups. *Nat. Commun.* **9**, 4353 (2018).

8. Koller, D. & Friedman, N. *Probabilistic Graphical Models: Principles and Techniques* (MIT Press, 2009).

9. Milholland, B., Auton, A., Suh, Y. & Vijg, J. Age-related somatic mutations in the cancer genome. *Oncotarget* **6**, 24627–24635 (2015).

10. Dunford, A. *et al.* Tumor-suppressor genes that escape from X-inactivation contribute to cancer sex bias. *Nat. Genet.* **49**, 10–16 (2017).

11. TCGA-Research-Network. Comprehensive genomic characterization defines human glioblastoma genes and core pathways. *Nature* **455**, 1061–1068 (2008).
